# Conceptualization and Implementation of the Central Information Portal on Rare Diseases: Protocol for a Qualitative Study

**DOI:** 10.2196/resprot.7425

**Published:** 2018-05-11

**Authors:** Svenja Litzkendorf, Tobias Hartz, Jens Göbel, Holger Storf, Frédéric Pauer, Ana Babac, Verena Lührs, Leena Bruckner-Tuderman, Franziska Schauer, Jörg Schmidtke, Lisa Biehl, TOF Wagner, J-Matthias Graf von der Schulenburg, Martin Frank

**Affiliations:** ^1^ Center for Health Economics Research Hannover Department of Insurance Leibniz University of Hanover Hannover Germany; ^2^ Clinical Cancer Registry of Lower Saxony Hannover Germany; ^3^ Medical Informatics Group University Hospital Frankfurt Frankfurt Germany; ^4^ Centre for Quality and Management in Healthcare Medical Association of Lower Saxony Hannover Germany; ^5^ Department of Dermatology Medical University Center of Freiburg Freiburg Germany; ^6^ Institute of Human Genetics Orphanet Deutschland Hannover Medical School Hannover Germany; ^7^ German Alliance of Chronic Rare Diseases Berlin Germany; ^8^ Frankfurt Center for Rare Diseases University Hospital Frankfurt Frankfurt Germany

**Keywords:** rare diseases, health information exchange, quality control, qualitative research

## Abstract

**Background:**

Recently, public and political interest has focused on people living with rare diseases and their health concerns. Due to the large number of different types of rare diseases and the sizable number of patients, taking action to improve the life of those affected is gaining importance. In 2013, the federal government of Germany adopted a national action plan for rare diseases, including the call to establish a central information portal on rare diseases (Zentrales Informationsportal über seltene Erkrankungen, ZIPSE).

**Objective:**

The objective of this study, therefore, was to conduct scientific research on how such a portal must be designed to meet the needs of patients, their families, and medical professionals, and to provide high-quality information for information seekers.

**Methods:**

We chose a 3-step procedure to develop a needs-based prototype of a central information portal. In the first step, we determined the information needs of patients with rare diseases, their relatives, and health care professionals by means of qualitative interviews and their content-analytical evaluation. On the basis of this, we developed the basic structure of the portal. In the second step, we identified quality criteria for websites on rare diseases to ensure that the information linked with ZIPSE meets the quality demands. Therefore, we gathered existing criteria catalogs and discussed them in an expert workshop. In the third step, we implemented and tested the developed prototypical information portal.

**Results:**

A portal page was configured and made accessible on the Web. The structure of ZIPSE was based on the findings from 108 qualitative interviews with patients, their relatives, and health care professionals, through which numerous information needs were identified. We placed particularly important areas of information, such as symptoms, therapy, research, and advisory services, on the start page. Moreover, we defined 13 quality criteria, referring to factors such as author information, creation date, and privacy, enabling links with high-quality information. Moreover, 19 users tested all the developed routines based on usability and comprehensibility. Subsequently, we improved the visual presentation of search results and other important search functions.

**Conclusions:**

The implemented information portal, ZIPSE, provides high-quality information on rare diseases from a central point of access. By integrating the targeted groups as well as different experts on medical information during the construction, the website can assure an improved search for information for users. ZIPSE can also serve as a model for other Web-based information systems in the field of rare diseases.

**Registered Report Identifier:**

RR1-10.2196/7425

## Introduction

### Finding Reliable Information on Rare Diseases as a Major Challenge

In Germany, an estimated 4 million people live with rare diseases [1]. Rare diseases, as defined by the Community Action Programme on Rare Diseases 1999-2003, are those with a prevalence of ≤1 per 2000 persons in the European Union [2]. Accordingly, of all known diseases, about 7000 can be considered rare. Even though causes and symptoms can widely vary, patients with rare diseases and their families often face similar challenges [3]. Among others, the affected people often lack reliable information about their own or their relatives’ disease due to unfamiliarity with information services or information retrieval systems. Additionally, for physicians who rarely encounter rare diseases in their daily practice, finding reliable information on diagnosis and treatment is a major challenge. Consequently, patients often wander from one doctor to another for years, until they receive a correct diagnosis and obtain access to specialized care. Therefore, in 2013, the federal government adopted a “National Action Plan on Rare Diseases” to improve patients’ health situation. Establishing an information system suitable for patients is one component of a broader set of measures to achieve this goal [4].

Currently, patients, their families, and health professionals have some difficulties in finding high-quality information on rare diseases [3]. Although there are plenty of websites, portals, and databases on rare diseases in the World Wide Web, including those on specific conditions and rare diseases in general, people do not know about these websites or struggle to find them in the vastness of the internet. Moreover, not every information website is suitable for different types of users and their specific needs. One of the largest databases on rare diseases in Europe, for instance, is Orphanet [5], which provides comprehensive information on a large number of rare diseases. Nonetheless, the information offered on this database meets the requirements of health professionals more than those of laypersons. Furthermore, there are some national and international information services, such as the National Organization for Rare Diseases in the United States and the Alliance of Chronic Rare Diseases in Germany [6,7]. However, the former as well as other foreign language offerings are not suitable for all people in Germany affected by or interested in knowing about the disease, due to language barriers. Yet, it is essential to understand disease information accurately. The latter only contains information on a small number of rare diseases, such that its usability is limited.

Apart from the aforementioned sources, centers for rare diseases and patient organizations often provide comprehensive and reliable information. Especially for patients and their relatives, the latter are important contact partners that help them access specialized care or offer advice on all questions relating to their disease. For people affected by a rare disease, physicians can be another important source of information [8-12]. However, apart from those who deal with these conditions on a regular basis, for instance, physicians working in centers for rare diseases, general practitioners, as well as specialists in private practice often lack such information.

### Developing a Central Information Portal on Rare Diseases

Therefore, this project aimed to conceptualize and implement a central information portal on rare diseases (ZIPSE) on the internet, through which people affected by a rare disease, their families, and relatives, as well as medical professionals can obtain access to high-quality information in German language. This should be done based on scientific methods and with the involvement of the different target groups. However, the portal’s editorial staff will not be generating the information provided on ZIPSE. Rather, it will identify, check, and link with ZIPSE, existing information sites on rare diseases, if they provide user-relevant information.

## Methods

### Evaluating the Information Needs of Patients, Relatives, and Health Care Professionals

To develop an information portal that suits the needs of patients, their relatives, and health professionals equally, over the entire course of the project, we aimed to integrate all target groups who may use the portal in the future. To ensure that the information provided on ZIPSE fulfills the needs of each target group as closely as possible, we initially evaluated the information needs of patients, relatives, and people working in the health care sector. Due to insufficient data on information needs in the field of rare diseases, we decided to use qualitative methods. For patients and their families, we developed an interview guide for eliciting information about their medical history, diagnostic processes, experience of living with the disease, and information searches. As many patients and their relatives ultimately join patient organizations, which can influence their awareness and knowledge of rare diseases, we asked them about their early experience of information gathering. To test whether the interview guide is suitable to identify individuals’ information needs, it was pretested with 2 patients and 1 relative. We subsequently adjusted the guide for those diagnosed before or shortly after birth, who could not remember their diagnostic paths.

To recruit a broad and balanced sample, we formed 11 groups of rare diseases at the beginning of this study, which represented a comprehensive variety of rare diseases. We planned to interview 6 patients or their family members in each group. Moreover, we conducted 10 interviews with patients who had waited for at least 10 years for diagnosis. Thus, we intended the sample to comprise 76 patients and close relatives. However, upon saturation of interview data, we found that a smaller sample was sufficient. Participants were recruited through the Freiburg Center for Rare Diseases at the University Medical Center Freiburg, University of Freiburg, Germany.

Our final sample involved a total of 68 participants, including 55 patients and 13 relatives (Table 1). Due to limited access to some patient groups, we could not ascertain the targeted number of interviews in all groups of diseases. However, as it became clear during the study that further interviews do not lead to further identification of information needs, no further recruitment was done.

To identify the information needs of health care professionals, we decided to conduct expert interviews. For this, we developed and pretested different structured interview guides for the different groups surveyed. In our sample, we considered physicians who are not related to centers of rare diseases and hence are inexperienced in information searches on rare diseases. These included general practitioners and medical specialists in private practice, as well as clinicians. Moreover, we interviewed medical technical assistants from in and out-patient care. Thus, experts in the care system, who are specialists in the field of rare diseases and guide patients or people suspected of suffering from a rare disease by the appropriate points of contact, comprised the interview sample. Apart from sociodemographic variables such as gender and age, other parameters integrated in sample selection included the nature of practice and geographical location. We did not strive for a certain sample size at the beginning of this study, but rather tried to reach theoretical saturation by conducting as many interviews as necessary with each sample group (physicians, medical technical assistants, and experts in rare diseases). The results of the interviews with the doctors were validated in a quantitative Delphi survey. Our final sample of health care professionals involved 28 physicians, 6 nurses, 4 guides, and 2 biologists.

We analyzed the interviews according to the structured content analysis method developed by Philipp Mayring [13]. Each audio recording was verbally transcribed and transferred to the MAXQDA (Verbi Software GmbH, Berlin) analysis software. Subsequently, 4 researchers examined the interviews independently, to mark all text passages providing information on people’s information needs.

Afterwards, an extensive system of categories using a deductive-inductive approach was developed. Therefore, the researchers processed 5 interviews to transfer the contents from the marked text passages into main- and sub-categories that represent detailed aspects of people’s information needs (inductive approach). Additionally, the researchers derived several categories (deductive approach) based on previous research on current information on rare diseases from the internet and published literature review [14-17]. These were integrated into the inductive categories stemming from the text (inductive approach). Then, we applied the system of categories to the rest of the marked text passages and modified or rather complemented it, if necessary.

Furthermore, we presented and discussed the information needs found in the interviews in 4 focus groups, to enable consensual validation. Participants of the focus groups were recruited chiefly from the initial study sample. In addition, some consultants from patient organizations were invited to participate. On the basis these results, the basic structure of the ZIPSE portal and information paths were developed.

### Defining the Quality Criteria for Websites on Rare Diseases

Defining the quality criteria for websites on rare diseases was the next step, as, owing to the large number of rare diseases, we planned to provide references to other internet sites instead of providing primary information. Therefore, we examined all quality certifications, catalogs of criteria, and recommendations for information on the Web existing in Germany, and compiled them in 1 conceptual map. In a workshop, several experts on quality of online information discussed this conceptual map with the project team to decide which quality criteria should be considered while linking information websites with the ZIPSE portal. Accordingly, we created a set of specific quality criteria on rare diseases.

Additionally, we conducted extensive research on existing German information websites on rare diseases to create a basic database for the subsequent inclusion of websites in accordance with the quality criteria. Therefore, we screened the internet for information websites on rare diseases using the German Orphanet list of rare diseases and their synonyms [18]. This list included all registered rare diseases. Several research assistants searched the most common browsers for all these diseases, and subsequently screened the first 20 entries offering information on the specific disease on which they sought information.

**Table 1:** Patient and relative demographics.

Characteristics in participants (N=68)		Statistics
Age (years), mean		50.5
**Gender, n (%)**		
	Female	45 (66)
	Male	23 (34)
**Rare disease, n (%)**		
	Genetic skin diseases	10 (15)
	Skeletal dysplasia	7 (10)
	Neuromuscular diseases	9 (13)
	Genetic eye diseases	4 (6)
	Connective tissue diseases	5 (8)
	Genetic kidney diseases	6 (9)
	Cystic fibrosis and pulmonary diseases	7 (10)
	Congenital blood formation disorders	4 (6)
	Immunodeficiency	7 (10)
	Congenital metabolic disorder	7 (10)
	Genetic diseases of the digestive tract	2 (3)
**Status, n (%)**		
	Patients	55 (81)
	Relatives	13 (19)

### Technical Implementation and Usability Tests

We used the information paths and functions developed based on the derived information needs to develop a prototype information portal. Thus, we set up a Uniform Resource Locator, on which essential elements could be activated and tested it concomitantly [19]. Initially, within the framework of the focus groups described above, we introduced patients and relatives, who had already participated in the interviews, to the preliminary version of the information portal, and they provided their opinion. Thus, we derived potential for improving the design of the home page of the website and of the search function within the portal. Subsequently, to check whether the derived information paths corresponded to people’s information needs, we conducted usability tests with patients, their relatives, and physicians. For this purpose, we asked the testers to browse through the portal and search for specific information on their disease while thinking aloud. Among the testers were 9 patients with rare diseases and 10 physicians, who did not participate in the interviews. As a result, authors could identify at which points of their search users may have problems in acquiring the information sought. Moreover, we measured their satisfaction with the use of the portal in personal discussions with the testers. A written survey was not conducted due to the small number of tests.

## Results

### Information Needs of Patients, Relatives, and Health Care Professionals

The findings of this study revealed a variety of information needs of patients with rare diseases and their families, which are published in detail elsewhere [20]. On the basis of content analysis, we derived various information areas relevant for the interviewees. First, we found that people’s information needs varied depending on the type and stage of illness. Shortly after diagnosis, the affected individuals reported the need for easily comprehensible and concise information, enabling an overall understanding of the disease, its causes, symptoms, and impact on their everyday life. Moreover, the participants stated that they wished for information on personal contacts of other patients or their relatives after they had received a diagnosis. At a later stage, people often mentioned the need for more detailed information on their disease. For example, they would like to know if there were any research efforts in which they or their relatives could participate. Knowledge about research on their illness is an important factor for many patients because it helps them cope with the illness and remain confident. Especially for those suffering from a severe rare disease that has not yet been researched intensively, this can be of enormous importance.

Content analysis showed that health care professionals’ needs partly overlapped with those of patients and their relatives. According to the respondents, doctors preferred to have basic information (eg, regarding prevalence or the course of the disease) about rare diseases before diagnosing the disease and assistance in the diagnostic process (eg, by obtaining information on special laboratories or specialized centers). Once the disease is diagnosed, the information needs to be shifted to the field of therapy coordination. The respondents assigned great importance to information about the counseling and care of patients with rare diseases as well as to a list of referred physicians and experts for further assistance. In addition, they deemed information on possibilities of exchanging experiences with other health professionals as well as medical education and training in the field of rare diseases necessary. The health care professionals also placed importance on research (eg, existing studies concerning disease progression). All the health care professionals stressed the importance of patient organizations and self-help groups. We verified the results of the interviews with the doctors in a Delphi survey.

We then used these information needs to further develop the ZIPSE information portal by placing these main information areas prominently on the ZIPSE start page, as well as by integrating them in the layout of the hit list display. Information on registered websites is assigned to these topics so that users can easily search those information topics. Clearly understandable icons, which are displayed on the hit list, indicate what information each individual information website covers.

### Quality Criteria for Websites on Rare Diseases

Overall, we identified 9 criteria catalogs and guidelines with recommendations for high-quality health information on the internet from a literature review. A total of 304 single items were extracted, which were reduced to 163 different criteria. Considering the large number of websites on rare diseases, quality criteria for the ZIPSE portal could not refer to the accuracy of all information offered, but to the quality of the information as well as of the preparation of information and of the website. Therefore, we selected criteria to assess how, by whom, and based on which sources the offered information had been collected; how data safety was to be addressed; and how users could contact the website operator. In a workshop with several experts from the field of medicine regarding high-quality online information, the conceptual map comprising 163 different criteria was reduced to a catalog containing 13 criteria, which was used for assessing information websites (Textbox 1). Among others, these criteria included data on the creation process, authors, sources, as well as creation and updating, data security, and declaration of evidence. Some of the criteria could be labeled as “essential criteria” (creation and updating date, data security, imprint, and contact information). Only if these essential criteria are fulfilled, a website will be displayed in the quality assured area of the portal. A criterion containing more than 1 term is deemed to be met when at least 1 term may be considered to be fulfilled. Websites that do not meet all essential criteria will be linked in a separate area. Users can actively request those, but we have to confirm that these websites do not meet the essential quality criteria. We published a detailed description relating to the adoption of quality criteria for websites providing medical information on rare diseases elsewhere [21].

We transferred all the identified quality criteria into a Web-based questionnaire that can be filled online either by the providers of information themselves or by the ZIPSE editorial team. Moreover, we conducted a comprehensive research on existing websites on rare diseases. We identified several hundred websites including those of patient organizations, research institutes, and care facilities and transferred them into a database containing information on the URL, the website provider, and the diseases. Subsequently, we contacted the website provider with a request to register or the editors of the ZIPSE portal registered their information themselves. The information websites were then visible and saved in the administration area of the portal’s home page. When website providers registered themselves, the editors of the ZIPSE portal reviewed all information for accuracy and integrity. If necessary, we corrected or completed data. Completely revised information websites that met the essential criteria were then activated, after which they were visible to people searching for related information in the hit list. Nonquality assured information websites were linked to a downstream area.

### Technical Implementation and Usability Tests

We set up a webpage on which we placed the basic developed framework of the ZIPSE portal [19]. During the course of the project, we added and evaluated various services, including a disease-specific search function as well as filter options. Through these features, users could search for information on a specific disease and filter search results by topic, information provider, or website features. Moreover, an administration interface was activated. In this interface, all data stored in the system (addresses, contact persons, information on the websites, and its quality aspects) could be managed. Thus, the quality of linked websites could be checked and documented recurrently.

To check whether the information paths, which were developed earlier, suited the target group’s specific needs, we conducted focus groups as well as usability tests. Initially, we created an extensive presentation to provide patients with rare diseases and their relatives an overall picture of the portal. Within the framework of focus group discussions, the participants reported high satisfaction regarding clarity, functionality, and comprehensibility. Nevertheless, they reported some points for improving the layout, such as the structural arrangement of the start-up page as well as the display of the hit list, among others. The results directly contributed to the further development and optimization of the ZIPSE portal. Elements on the home page, which were less important to the testers such as the offer to recommend or register a website, can now be found at the bottom of the start-up page, whereas main information areas as well as a mapping of care facilities were placed at a more prominent location. Additionally, new, self-explanatory pictograms for the hit list were created. In the usability tests with patients and physicians, it followed that several aspects could be revealed, which we subsequently revised, including a larger representation of the search field, a clearer presentation of the filter options, and an unambiguous representation of icons and images.

**Zentrales Informationsportal über seltene Erkrankungen (ZIPSE) quality criteria.**

**Creation process**

- Do you perform systematic (literature) research for information creation on your home page? If yes, please describe this process.
- Are experts involved in information creation? If yes, which?
- Is the process of building information on the website documented? If yes, what does this documentation look like? (Please describe)
- Do you illustrate the information building process for your users? If yes, please describe the presentation and name the respective Uniform Resource Locator (URL).

**Authors**

- General information (names and qualifications) about the authors has been mentioned.
- Other persons, who contributed to developing information, are mentioned.
- Contents authored by users have been labeled and equipped with a user name.

**Sources**

- Do you provide self-created information?
- If no, do you mention external sources?

**Creation and updating (essential criterion)**

- The creation date of the information has been mentioned.
- The updating date of the information has been mentioned.

**Data security (essential criterion)**

- By means of a privacy policy, do you inform the user about the usage, storage, and disclosure of personal data?
- Do you inform the user in a prominent position about the storage of personal data for internal usage (eg, research) with an analysis tool? Does the user have the option to disagree?
- Does the user need to agree actively to the disclosure of personal data to third parties?

**Declaration of the evidence**

- Is all medical information evidence-based, whereby it is discernible on which basis points are made (eg, studies and expert statements)?
- Do you show the user references to limits of the evidence respectively name more evidence needs?

**Marking of conflicts of interests**

- Advertisements have been marked as such clearly.
- Sponsors have been named.
- Targets and purposes of the home page have been published clearly (eg, commercial interest).
- The funding (except from self-financing) source has been published.
- Conflicts of interests have been declared.

**Consideration of target group**

- Information is target-group specific.
- It is discernible to whom the information is addressed (eg, patients or doctors)?

**Evaluation**

- An archive of former or changed contents exists.
- The accuracy of all the information has been checked consistently.

**Review process**

- Do you have an internal review process (content quality assessment) for the evaluation of the contents?
- If yes, please describe it.

**Characteristics of the website (low-barrier)**

- Did you check the website for accessibility through a Barrierefreie Informationstechnik-Verordnung-Test (better: barrier-free information technology regulation test?)? If yes, how many points has the website achieved on this test?
- Is the font size of the website adjustable?
- Do you consider persons with color deficiency in your coloration?
- Can the main menu be accessed without a mouse?
- Is the information available in a simple language (eg, according to the rules of the network simple language)?
- Is the information website available in several languages?
- It is possible to subscribe to a newsletter?
- Is the information available in a printable version?
- Are multimedia contents available (eg, videos and photos)?

**Imprint (essential criterion)**

Does the imprint contain the following information:

- Name and address of the publisher
- Email address of the publisher
- Declaration of the commercial register, the register of associations, etc, in which the provider is registered, and the respective registration number)

**Contact (essential criterion)**

- Users can provide feedback or contact the operator.
- A contact sheet is easy to access.

**Figure 1:**
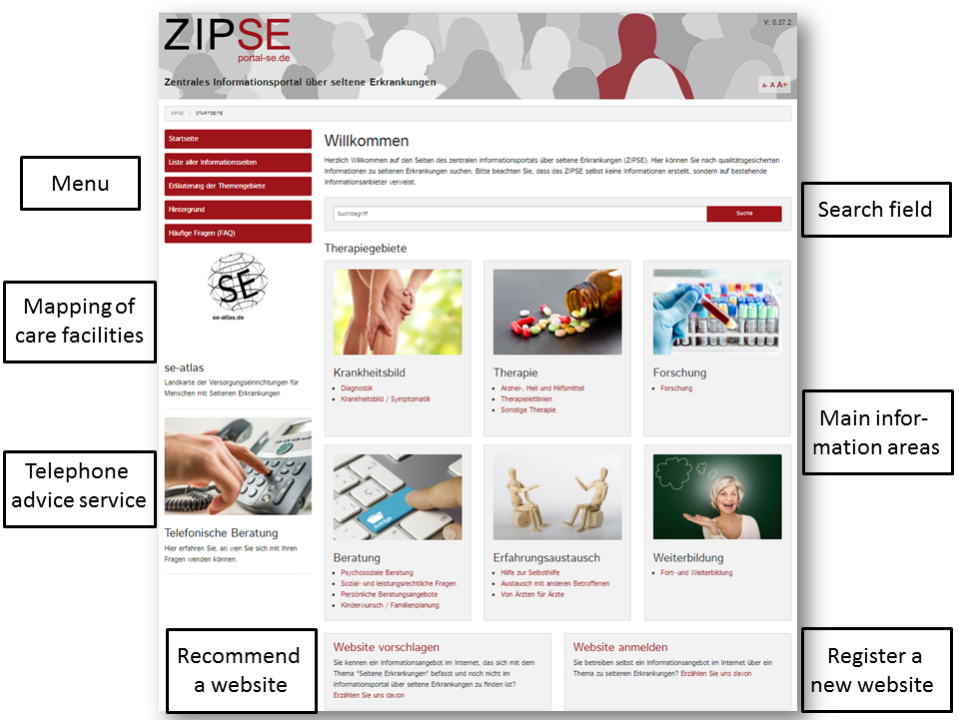
Layout of the Zentrales Informationsportal über seltene Erkrankungen (ZIPSE) start-up page.

**Figure 2:**
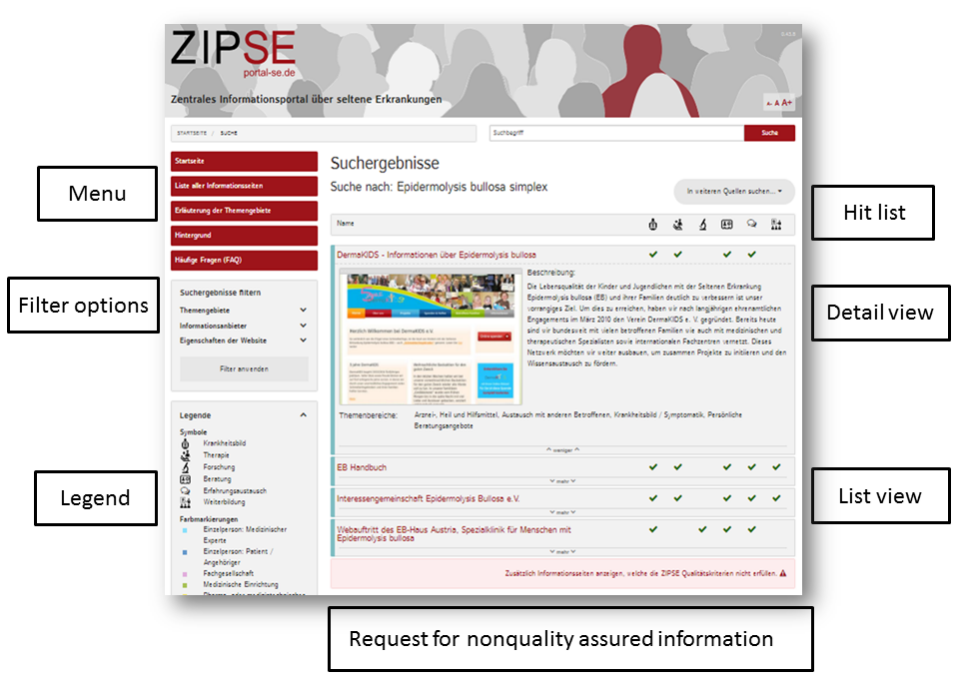
Layout of the Zentrales Informationsportal über seltene Erkrankungen (ZIPSE) hit list page.

### Prototypical Implementation of the Central Information Portal on Rare Diseases

As a result of the procedure described above, we created a functioning information portal. Figure 1 shows the structure of the ZIPSE start-up page. Below the ZIPSE header, users can find the search field in which they can conduct a disease-related search. The menu is displayed in the upper left corner, under which a link to the se-atlas, a mapping service for care facilities on rare diseases, as well as information about telephone advice has been provided. At the bottom of the website, users can find the option to either propose the addition of an information website on rare diseases by providing its name and contact persons, or to register themselves in case they operate a website on a rare disease. Above this option, a selection of different information areas of particular importance regarding the needs identified earlier has been displayed prominently.

When searching for a disease using the search field, a list of linked information websites appears (Figure 2). In this list, all the relevant websites on that specific disease, which have been registered and reviewed positively regarding the essential quality criteria, are presented alphabetically. Next to the name of each information website, an overview of the information areas covered by each site appears (represented by green checkmarks). Moreover, on the left side, users can filter all hits regarding further information areas (which might not have been considered directly in the hit list, but still are relevant), the type of provider, as well as characteristics of the website (such as accessibility and multilingualism). Clicking on one of the matches produces a detailed view of the website. This includes a screenshot of the webpage’s start-up page, as well as a short description of the contents of this site. The users then have the option to be forwarded to the website by clicking on its image. Information websites that do not meet the essential quality criteria can be requested at the bottom of the hit list. By clicking the corresponding button, these are shown in the hit list and are clearly marked.

## Discussion

### Principal Findings

During the course of the project, we developed and implemented a prototype of a central information portal on rare diseases that fulfills the needs of patients, their relatives, physicians, and other health care professionals. Currently, 720 information websites have registered on the portal. About half of the websites meet the essential quality criteria, whereas the other half can be found in the nonquality assured area of the portal. A total of 239 websites refer to genetically caused diseases (239/720, 33.1%). Another 92 websites focus on neurological conditions (92/720, 12.7%). This is followed by 72 websites on neoplastic diseases (72/720, 10.0%) as well as 40 websites on developmental defects during embryogenesis (40/720, 5.5%). All other disease groups are in the lower single-digit percentage range.

The information needs identified through qualitative interviews were in line with different international studies [14-17] as well as with information offerings on a random sample of existing websites on rare diseases, selected from the ZIPSE database. On the ZIPSE portal, users can search for information on rare diseases from this central point of access. There also lies the crucial benefit over other online offerings on rare diseases, which are often widely dispersed over the internet. To switch from one source to another can be challenging for information seekers. ZIPSE combines all the varying kinds of information websites at one central point. Moreover, all the available information on ZIPSE can be easily filtered by topics that have been shown to be particularly important or by the type of provider as well as by characteristics of the website. Thus, the compiled high-quality information will be more accessible to the interested or affected people.

To offer added value to people with rare diseases, continuous maintenance and optimization of the information portal and its structures and services is of utmost importance. Not only must existing links and contents be kept up to date, but other information websites, including websites from English-speaking countries, must also be identified and integrated within the portal. Especially for people with diseases for which little information is available in German, such information in English could be very useful. Therefore, we will maintain, update, and continuously develop ZIPSE. However, to sustain the availability of ZIPSE, a major challenge for the near future would be to find funding sources. To make sure that all work continues, we are constantly developing different solutions for sustainable funding after the end of this publicly funded project.

By compiling information on rare diseases at one central point of access, in the future, people can identify gaps in knowledge about specific diseases more easily. One can infer that there is insufficient information on all rare diseases, especially on very rare diseases, where only little research has been carried out owing to financial restrictions as well as small numbers of available patients. Without research, no knowledge and information can be generated. This explains why for many (very) rare diseases only little or no information is available online. Addressing these knowledge gaps could be an important task for future studies.

Our concept of a central information portal on rare diseases could be useful as a model for other information providers in the field of rare diseases, for the development of similar information systems. Even though there is a range of other information systems providing information on rare diseases to different target groups, this is the first one that was developed by using extensive scientific methods and integrating all target groups in its development. Due to this study’s underlying scientific approach regarding the collection of people’s information needs and definition of quality criteria, as well as the involvement of patients, their relatives, and physicians at all stages of the project, one can assume a high target group–specific alignment that could be transferred to other systems.

### Limitations

Due to the limited financial and personnel resources in this publicly funded research project, some of the ideas regarding the structure and function of the information portal could not be fully developed. These include, for example, the establishment of a newsletter that informs users about newly included information sites on specific diseases or issues when requested. This could be a task for future operators of the website.

### Conclusions

Dealing with the various challenges arising from rare diseases has become an important task for most health care systems. Especially, the gaps in knowledge and the uncertain quality of information pose challenges for the establishment of networks of information infrastructures. Even though there is information on many rare diseases, it is often insufficiently known and used due to low visibility. Establishing a central information portal like ZIPSE makes the existing but widely dispersed information accessible to the various groups of people dealing with rare diseases.

For patients and their families, this offers an opportunity for easy access to extensive information on topics that are important to them, such as therapy, social and legal issues, and self-help. For doctors and other medical professionals, the ZIPSE portal can help to accelerate the diagnostic process and improve patient care by providing information on rare disease diagnostics, therapy, and specialized care facilities. Therefore, bundling high-quality information at one central access point can improve people’s health care sustainably. In the future, it will be easy to find trustworthy information for people living with a rare disease by using the ZIPSE portal. Furthermore, with reference to professional caregivers, reducing uncertainties in diagnostics and therapy could prevent the overuse, underuse, and misuse of information in the health care sector. Moreover, the ZIPSE portal can help raise awareness about rare diseases in general. One of the current challenges concerning rare diseases is not only missing information but also the lack of awareness about them. Along with closing gaps in people’s knowledge, the ZIPSE portal can help sensitize people regarding rare diseases.

## Abbreviations

BMG: Federal Ministry of Health Germany
ZIPSE: Central Information Portal on Rare Diseases
