# Metadata Correction: Conceptualization and Implementation of the Central Information Portal on Rare Diseases: Protocol for a Qualitative Study

**DOI:** 10.2196/11248

**Published:** 2018-09-19

**Authors:** Svenja Litzkendorf, Tobias Hartz, Jens Göbel, Holger Storf, Frédéric Pauer, Ana Babac, Verena Lührs, Leena Bruckner-Tuderman, Franziska Schauer, Jörg Schmidtke, Lisa Biehl, TOF Wagner, J-Matthias Graf von der Schulenburg, Martin Frank

**Affiliations:** 1 Center for Health Economics Research Hannover Department of Insurance Leibniz University of Hanover Hannover Germany; 2 Clinical Cancer Registry of Lower Saxony Hannover Germany; 3 Medical Informatics Group University Hospital Frankfurt Frankfurt Germany; 4 Centre for Quality and Management in Healthcare Medical Association of Lower Saxony Hannover Germany; 5 Department of Dermatology Medical University Center of Freiburg Freiburg Germany; 6 Institute of Human Genetics Orphanet Deutschland Hannover Medical School Hannover Germany; 7 German Alliance of Chronic Rare Diseases Berlin Germany; 8 Frankfurt Center for Rare Diseases University Hospital Frankfurt Frankfurt Germany

The corresponding author of the paper “Conceptualization and Implementation of the Central Information Portal on Rare Diseases: Protocol for a Qualitative Study” (JMIR Res Protoc 2018;7(5):e112), made a mistake in the final stage of proofreading. The academic degrees of Leena Bruckner-Tuderman, Jörg Schmidtke, TOF Wagner, and Franziska Schauer were incorrectly listed as “PhD”. Instead, the degrees for all four of these authors should be “Dr med”, which represents an academic degree according to the German academic system.

The correction will appear in the online version of the paper on the JMIR website on September 6, 2018, together with the publication of this correction notice. Because this was made after submission to PubMed, Pubmed Central, and other full-text repositories, the corrected article also has been re-submitted to those repositories.

